# Reduced Expression of miR-200 Family Members Contributes to Antiestrogen Resistance in LY2 Human Breast Cancer Cells

**DOI:** 10.1371/journal.pone.0062334

**Published:** 2013-04-23

**Authors:** Tissa T. Manavalan, Yun Teng, Lacey M. Litchfield, Penn Muluhngwi, Numan Al-Rayyan, Carolyn M. Klinge

**Affiliations:** Department of Biochemistry and Molecular Biology, Center for Genetics and Molecular Medicine, University of Louisville School of Medicine, Louisville, Kentucky, United States of America; Wayne State University School of Medicine, United States of America

## Abstract

**Introduction:**

The role of miRNAs in acquired endocrine-resistant breast cancer is not fully understood. One hallmark of tumor progression is epithelial-to-mesenchymal transition (EMT), characterized by a loss of cell adhesion resulting from reduced E-cadherin and increased cell mobility. miR-200 family members regulate EMT by suppressing expression of transcriptional repressors ZEB1/2. Previously we reported that the expression of miR-200a, miR-200b, and miR-200c was lower in LY2 endocrine-resistant, mesenchymal breast cancer cells compared to parental, endocrine sensitive, epithelial MCF-7 breast cancer cells. Here we investigated the regulation of miR-200 family members and their role in endocrine-sensitivity in breast cancer cells.

**Results:**

miR-200 family expression was progressively reduced in a breast cancer cell line model of advancing endocrine/tamoxifen (TAM) resistance. Concomitant with miR-200 decrease, there was an increase in ZEB1 mRNA expression. Overexpression of miR-200b or miR-200c in LY2 cells altered cell morphology to a more epithelial appearance and inhibited cell migration. Further, miR-200b and miR-200c overexpression sensitized LY2 cells to growth inhibition by estrogen receptor (ER) antagonists TAM and fulvestrant. Knockdown of ZEB1 in LY2 cells recapitulated the effect of miR-200b and miR-200c overexpression resulting in inhibition of LY2 cell proliferation by TAM and fulvestrant, but not the aromatase inhibitor exemestane. Demethylating agent 5-aza-2′-deoxycytidine (5-aza-dC) in combination with histone deacetylase inhibitor trichostatin A (TSA) increased miR-200b and miR-200c in LY2 cells. Concomitant with the increase in miR-200b and miR-200c, ZEB1 expression was decreased and cells appeared more epithelial in morphology and were sensitized to TAM and fulvestrant inhibition. Likewise, knockdown of ZEB1 increased antiestrogen sensitivity of LY2 cells resulting in inhibition of cell proliferation.

**Conclusions:**

Our data indicate that reduced miRNA-200b and miR-200c expression contributes to endocrine resistance in breast cancer cells and that the reduced expression of these miR-200 family members in endocrine-resistant cells can be reversed by 5-aza-dC+TSA.

## Introduction

EMT (epithelial-to-mesenchymal transition) is a hallmark of metastatic cancer [1]. EMT is induced by activation of signaling pathways, *e.g*., TGF-β, Wnt, and Notch [2], [3]. EMT is characterized by loss of the epithelial marker E-cadherin resulting from gene methylation or repression by upregulation of transcriptional repressors Zinc finger E-box binding homeobox domain proteins ZEB1 (also known as TCF1 or δEF1) or ZEB2 (also known as SIP1), Snail1/2, and TWIST [4], [5], [6], [7]. As a result of the reduction in E-cadherin and cell-cell interactions, cells acquire a mesenchymal phenotype distinguished by the expression of vimentin and N-cadherin [8]. Members of the miR-200 family and miR-221/222 are implicated in EMT and metastasis [9]. Reduced expression of these miRNAs has been reported in metastatic breast cancer [10], [11], [12]. Other miRNAs with roles in maintaining the epithelial phenotype of cells include miR-203, miR-205, miR-99a, miR-99b, miR-130a and miR-34a/b/c [13], [14], [15], [16], [17].

The link between the development of endocrine-resistance and EMT in breast cancer is still not clearly understood. While most patients with estrogen receptor α (ERα)-positive tumors initially benefit from endocrine therapies, *e.g*., tamoxifen (TAM), and aromatase inhibitors, *e.g*., exemestane, resistance develops in ∼40–50% of these patients [18]. ERα expression promotes the differentiation of mammary epithelial cells and opposes EMT: thus, a loss of ERα expression, *e.g*., by upregulation of miR-206 that targets ERα, is associated with EMT [17]. Studies have shown that endocrine-resistance endows metastatic properties to cells. For example, some endocrine resistant cells and tumors acquire activation of the β-catenin pathway and induction of Snail and TWIST that contribute to EMT [19], [20], [21]. A few studies have shown that miRNAs have a role in conferring endocrine-resistance which subsequently leads to induction of EMT and metastasis. Re-expression of miR-375 restored TAM sensitivity and reverted EMT in TAM-resistant MCF-7 breast cancer cells [22]. Prolonged growth of MCF-7 cells as mammospheres induced EMT and resistance to TAM [23]. Notably, these cells exhibited higher expression of miR-221/222 and reduced expression of miR-200c, miR-203, and miR-205 [23]. Overexpression of miR-221/222 has been shown to be essential for acquired resistance of breast cancer cells to antiestrogens and to activate ß-catenin and inhibit TGF-ß signaling in fulvestrant-resistant breast cancer cells [24].

The miR-200 family of miRNAs are transcribed from two chromosomal locations: miR-200b, miR-200a, and miR-429 are located on chromosome 1p36; while miR-200c and miR-141 are located on chromosome 12p13 [14]. The miR-200bc/429 cluster differs from the miR-200a/141 cluster by the fourth nucleotide (U to C) in the seed region; thus, they regulate different target genes in breast cancer [25]. Reduced expression of the miR-200 family has been observed in breast, ovarian, endometrial, lung and gastric cancer [26]. Many studies have identified an inverse relationship between the expression of the miR-200 family and its targets ZEB1/2 in cells [27], [28], [29], [30], [31]. We recently reported increased expression of ZEB1 protein and loss of its target E-cadherin in an endocrine-resistant, ERα+ breast cancer cell line LY2 compared to the parental MCF-7 cell line [32]. LY2 cells had undetectable levels of miR-200 family members compared to MCF-7 cells, suggesting a role for miR-200 in TAM/endocrine-resistance and loss of ZEB1 repression.

Here we examined the expression of miR-200a, miR-200b, and miR-200c and their regulation by estradiol (E_2_) and 4-hydroxytamoxifen (4-OHT), an active TAM metabolite, in a panel of ERα-positive breast cancer cell lines derived from MCF-7 endocrine-sensitive cells representing a cellular model of progression towards endocrine/TAM-resistance. We report that transient overexpression of miR-200b and miR-200c in LY2 cells sensitized the cells to inhibition by antiestrogens TAM and fulvestrant (ICI 182,780). Further, overexpression of miR-200b and miR-200c also altered morphology of cells from a mesenchymal to an epithelial phenotype and reduced ZEB1/2 mRNA expression. Knockdown of ZEB1 increased sensitivity of LY2 cells to TAM and fulvestrant. Our results indicate a role for loss of miR-200 family members and increased ZEB1 in conferring resistance to antiestrogens in breast cancer. We suggest that in addition to being a biomarker for EMT, reduced miR-200 expression may serve as a prognostic marker in acquired endocrine resistance.

## Materials and Methods

### Cell Culture

MCF-7 human breast cancer cells were purchased from ATCC (Manassas, VA, USA) and maintained in IMEM supplemented with 5% fetal bovine serum and 1% penicillin/streptomycin (Invitrogen, Carlsbad, CA, USA) [14]. LCC1 (E2-resistant, TAM-sensitive derivatives of MCF-7 cells [33]); LCC2, LCC9, and LY2 (E2-independent, TAM, other selective ER modulator (SERM)-resistant derivatives of MCF-7 cells) were graciously provided by Dr. Robert Clarke, Georgetown University, Washington, DC, USA [33], [34], [35], [36]. Prior to treatment, the medium was replaced with phenol red-free IMEM supplemented with 5% dextran-coated charcoal-stripped FBS (DCC-FBS) and 1% penicillin/streptomycin (stripped medium) for 48 h (referred to as ‘serum-starving’).

### Chemicals

E_2_, 4-OHT, and exemestane (aromatase inhibitor) were purchased from Sigma-Aldrich (St. Louis, MO). ICI 182,780 (fulvestrant) was from Tocris (Ellisville, MO, USA). Cells were treated with ethanol (EtOH, the vehicle control, 0.01% final volume), 10 nM E_2_, or 100 nM 4-OHT, or other concentrations, for 6 h, as indicated. Where indicated, LY2 cells were treated with 2.5 µM 5-aza-2′-deoxycytidine (Sigma-Aldrich) alone or in combination with 100 ng/µl Trichostatin A (TSA, Sigma-Aldrich) for 72 h, with TSA added 16 h prior to RNA isolation [37].

### RNA Isolation and Quantitative Real-Time-PCR (qPCR) for miRNA and mRNA Expression

miRNA-enriched total RNA was extracted from MCF-7 and LY2 cells using the miRNA isolation kit (Exiqon, Woburn, MA, USA). The quality and quantity of the isolated RNA was analyzed using a NanoDrop spectrophotometer and Agilent Bioanalyzer. cDNA was synthesized using the miRCURY LNA™ first strand cDNA synthesis kit (Exiqon) and qPCR was performed using the miRCURY LNA™ SYBR Green master mix (Exiqon) using the miRNA primer sets for miR-200a, miR-200b, or miR-200c (Exiqon). SNORD38B and 5SRNA were used for normalization of miRNA expression. Analysis and fold change was determined using the comparative threshold cycle (Ct) method. The change in miRNA expression was calculated as fold-change, *i.e.*, relative to EtOH-treated (control).

For mRNA expression, the High Capacity cDNA Reverse Transcription kit (Life Technologies, Carlsbad, CA, USA) was used to reverse transcribe total RNA using random hexamers. qPCR for *ZEB1* was performed using SYBR green in the ABI PRISM 7900 SDS 2.1 (Life Technologies) using relative quantification. The sequence of the primers for ZEB1, ZEB2, E-cadherin, Vimentin and TGF-ß are described in [14]. GAPDH or 18S were used as the endogenous controls. Analysis and fold differences were determined using the comparative CT method. Fold change was calculated from the ΔΔCT values with the formula 2^−ΔΔCT^ and data are relative to EtOH-treated cells.

### Transient Transfection

MCF-7 or LY2 cells were transfected with either miRNA inhibitors (Anti-miR^TM^s, Ambion, Austin, TX) or microRNA precursors (Pre-miR^TM^s, Ambion) for miR-200b or miR-200c using Lipofectamine RNAiMAX reagent (Invitrogen). Negative controls were *mir*Vana™ miRNA inhibitor, Negative Control #1; or Pre-miR™ negative control (Ambion). After 1, 5, or 11 d, RNA was isolated (as described above) to confirm knockdown or overexpression of miR-200b or miR-200c. For ZEB1 knockdown studies, LY2 cells were transfected with Silencer® select siRNA against ZEB1 (siZEB1 cat#: 4392420; clone 1: s229971; clone 2: s229972) or a negative control, Silencer® Negative Control #1 siRNA (Ambion) for 48 h prior to RNA isolation.

### MTT Assay

MCF-7 or LY2 cells were grown in 96 well plates. Following transfection with anti-miRs or pre-miRs, or controls as above, respectively for 24 h or 5 days, cells were treated with vehicle control EtOH, 10 nM E_2_, 100 nM 4-OHT, or 100 nM fulvestrant (ICI 182,780) for 6 days. 20 µl of Cell Titer 96^R^ Aqueous One solution (Promega, Madison, WI, USA) was added to the wells and absorbance was read at 490 nm using a spectrophotometer (Spectromax M12). Each treatment was performed in quadruplicate within each experiment and experiments were repeated three times for statistical evaluation.

### BrdU Assay

LY2 cells were grown in 96 well plates. The cells were seeded at a density of 2000 cells/well in 96 well plates and were incubated ∼16 h (overnight) in growth medium prior to transfection with negative control siRNA (Invitrogen cat no. 4390843) or two different clones of Stealth siRNA for ZEB1 (Invitrogen, Carlsbad, CA, USA) using Lipofectamine (Invitrogen). 24 h after transfection, the cells were treated with 100 nM or 1 µM 4-OHT or fulvestrant; or with 100 nM exemestane in phenol red-free IMEM +5% DCC-stripped serum. Cells were incubated for 48 h at 37C° and 5% CO_2_. Cell proliferation was determined by measuring BrdU incorporation using an ELISA kit from Roche Applied Science (cat. 11647229001, Indianapolis, IN, USA) according to the manufacturer’s instructions. Each treatment/transfection was performed in quadruplicate. Absorbance readings in EtOH treated cells were used as control to evaluate relative BrdU incorporation as an index of cell proliferation.

### Whole Cell and Nuclear Lysate Preparation for Western Blotting

Whole cell lysates were prepared and western blots were performed as described in [32]. Nuclear extracts (NE) were prepared using the NE-PER kit from Thermo Scientific (Rockford, IL, USA). Protein concentrations were determined by BioRad DCC protein assay (Hercules, CA, USA).

### Antibodies and Reagents

Antibodies were purchased as follows: E-cadherin (Cell Signaling, Danvers, MA, USA), vimentin and N-cadherin (Santa Cruz Biotechnology, Santa Cruz, CA), Slug/SNAI2 (Millipore, Billerica, M, USA), and ß-actin (Sigma-Aldrich). The ZEB1 antibody was a kind gift from Dr. Douglas S. Darling (University of Louisville School of Dentistry). Chemiluminescent bands on the PVDF membranes were visualized on a Kodak Image Station 4000R Pro using Carestream Molecular Imaging software (New Haven, CT, USA).

### Microscopy Images

LY2 cells were untransfected or transfected with a negative control, pre-miR-200b, or pre-miR-200c for 48 h (see above). Images were captured using a digital microscope (EVOS, AMG, Bothell, WA, USA) at a magnification of 20× and 400 µm scale.

### Wound Healing Assay

LY2 cells were plated in six-well plates in phenol red-free IMEM +5% DCC-FBS for 24 h. Cells were transfected with a negative control or with pre-miR-200b or pre-miR-200c (see above) for 24 h. Cells were wounded by scratching with a p200 pipette tip and then washed with medium to remove displaced cells. Images were captured at 20X magnification using an EVOS microscope and NIH Image J software was used to analyze the percent of wound area at each time point. Values were averaged from four separate readings at each time point. Chi square test was performed using Excel.

### Statistical Analysis

Statistical evaluations were performed using GraphPad PRISM. Student’s t-test was used to compare control and treatment values. P-values indicate statistical significance.

## Results

### Expression of miR-200 Family in MCF-7, LCC1, LCC2, LCC9 and LY2 Human Breast Cancer Cells

Microarray analysis of miRNA expression revealed low expression of miR-200a, miR-200b, and miR-200c in LY2 endocrine-resistant breast cancer cells compared to MCF-7 endocrine-sensitive breast cancer cells [32]. To follow up on this initial observation, the expression of miR-200a, miR-200b and miR-200c was measured by qPCR in a panel of human breast cancer cell lines, *i.e*., LCC1, LCC2 and LCC9 cells that were derived from the parental MCF-7 cell line by propagation first as a xenograft in ovariectomized, athymic nude mice (LCC1), and then in long-term culture with tamoxifen (LCC2) or fulvestrant (LCC9) [33]. LY2 tamoxifen/fulvestrant-resistant human breast cancer cells were independently derived from MCF-7 cells by continuous cultivation in medium containing increasing concentrations of a drug precursor to raloxifene: LY 117018 [36]. LY2 are cross-resistant to TAM, raloxifene, fulvestrant and are ERα positive, although ERα protein expression is lower than MCF-7 cells [32], [38]. These cells represent a model of the progression of breast cancer cells towards TAM/endocrine-resistance [38].

**Figure 1:**
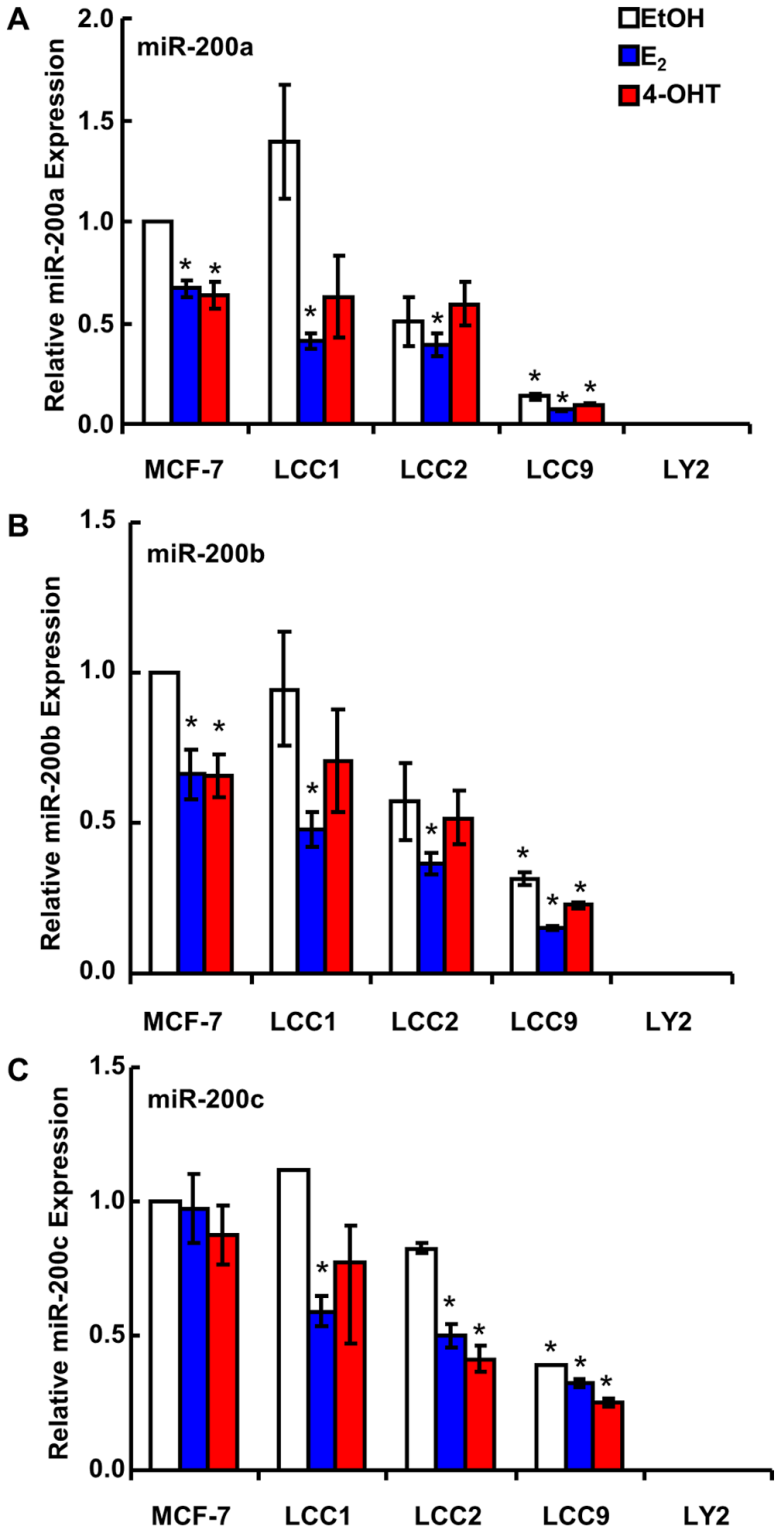
Expression of miR-200 family members in MCF-7, LCC1, LCC2, LCC9 and LY2 cells. Cells were serum-starved for 48 h and then treated with vehicle control EtOH, 10 nM E_2_, or 100 nM 4-OHT for 6 h. miR-200 expression was quantified by qPCR. Values are the mean ± SEM of 3–4 experiments and are expressed as fold relative to EtOH-treated MCF-7 expression for each miRNA. *p<0.05 *versus* MCF-7 EtOH-treated.

Basal miR-200 and the effect of E_2_ and 4-OHT on miR-200 expression was examined by qPCR in the cell lines described above (Fig. 1 and Fig. S1). There was no difference in basal miR-200a, miR-200b, or miR-200c expression between MCF-7 and LCC1 cells. However, LCC2 and LCC9 cells had lower miR-200 family member expression compared to MCF-7 cells. 10 nM E_2_ and 100 nM 4-OHT significantly decreased miR-200a and miR-200b expression in MCF-7 cells, but had no effect on miR-200c expression. Similarly, E_2_ significantly decreased the expression of miR-200a, miR-200b, and miR-200c in estrogen-independent, but TAM-sensitive LCC1 cells. However, there was no effect of E_2_ and 4-OHT on the expression of miR-200 family in LCC2, LCC9 and LY2, reflecting their endocrine resistance. LY2 cells had undetectable levels of miR-200 family expression. This is the first report of 4-OHT regulation of miR-200 family expression in LCC1, LCC2, LCC9 and LY2 cells. We and others previously reported that E_2_ reduces miR-200 family expression in MCF-7 cells [32], [39].

### E_2_ and 4-OHT Regulate ZEB1 in MCF-7, LCC1, LCC2, LCC9 and LY2 Human Breast Cancer Cells

**Figure 2:**
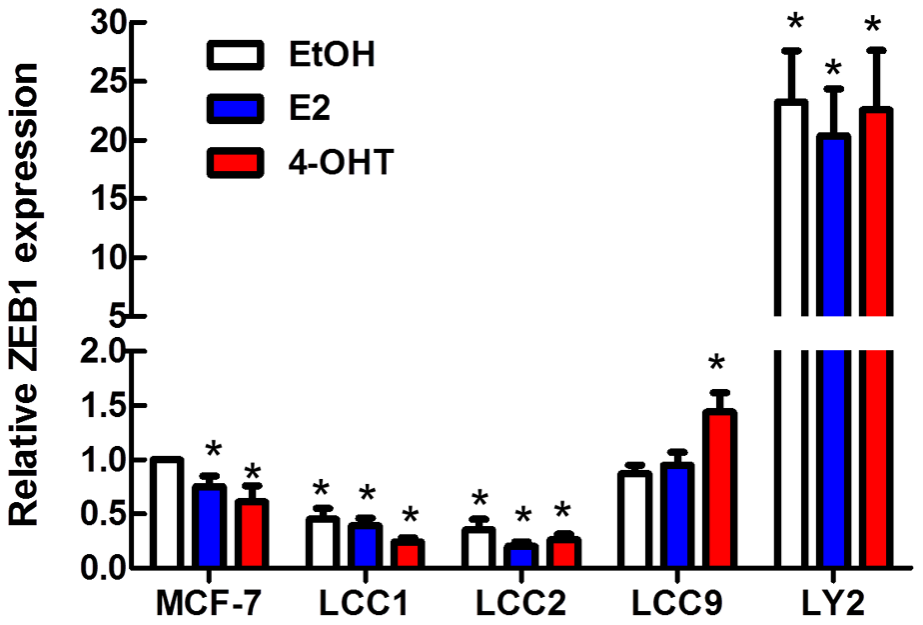
ZEB1 mRNA expression in MCF-7, LCC1, LCC2, LCC9 and LY2 cells. Cells were serum-starved for 48 h and treated with EtOH (vehicle control), 10 nM E_2_, or 100 nM 4-OHT for 6 h. ZEB1 expression was quantified by qPCR. Values are the mean ± SEM of 3 experiments and are expressed as fold relative to EtOH-treated MCF-7. *p<0.05 *versus* EtOH-treated MCF-7.

miR-200 family members repress ZEB1 expression at the mRNA and protein levels [14], [26], [27], [28]. Basal ZEB1 expression was lower in LCC1 cells compared to MCF-7 cells (Fig. 2A). As previously reported, ZEB1 expression was higher in LY2 compared to MCF-7 cells [32]. Notably, there is an inverse relationship between the expression of miR-200 family and ZEB1 in LY2 cells (compare Fig. 1 and 2). ZEB1 expression was lower in LCC1 and LCC2 than in MCF-7 cells. E_2_ and 4-OHT decreased the expression of ZEB1 in MCF-7 cells (Fig. 2). There was no significant effect of either E_2_ or 4-OHT on ZEB1 expression in LCC1 or LCC2 cells. E_2_ did not affect ZEB1 expression in LCC9 or LY2 cells; however, 4-OHT increased ZEB1 expression in tamoxifen-resistant LCC9 cells (Fig. 2).

### Overexpression of miR-200b or miR-200c in LY2 Cells Enhances Inhibition by 4-OHT or Fulvestrant

**Figure 3:**
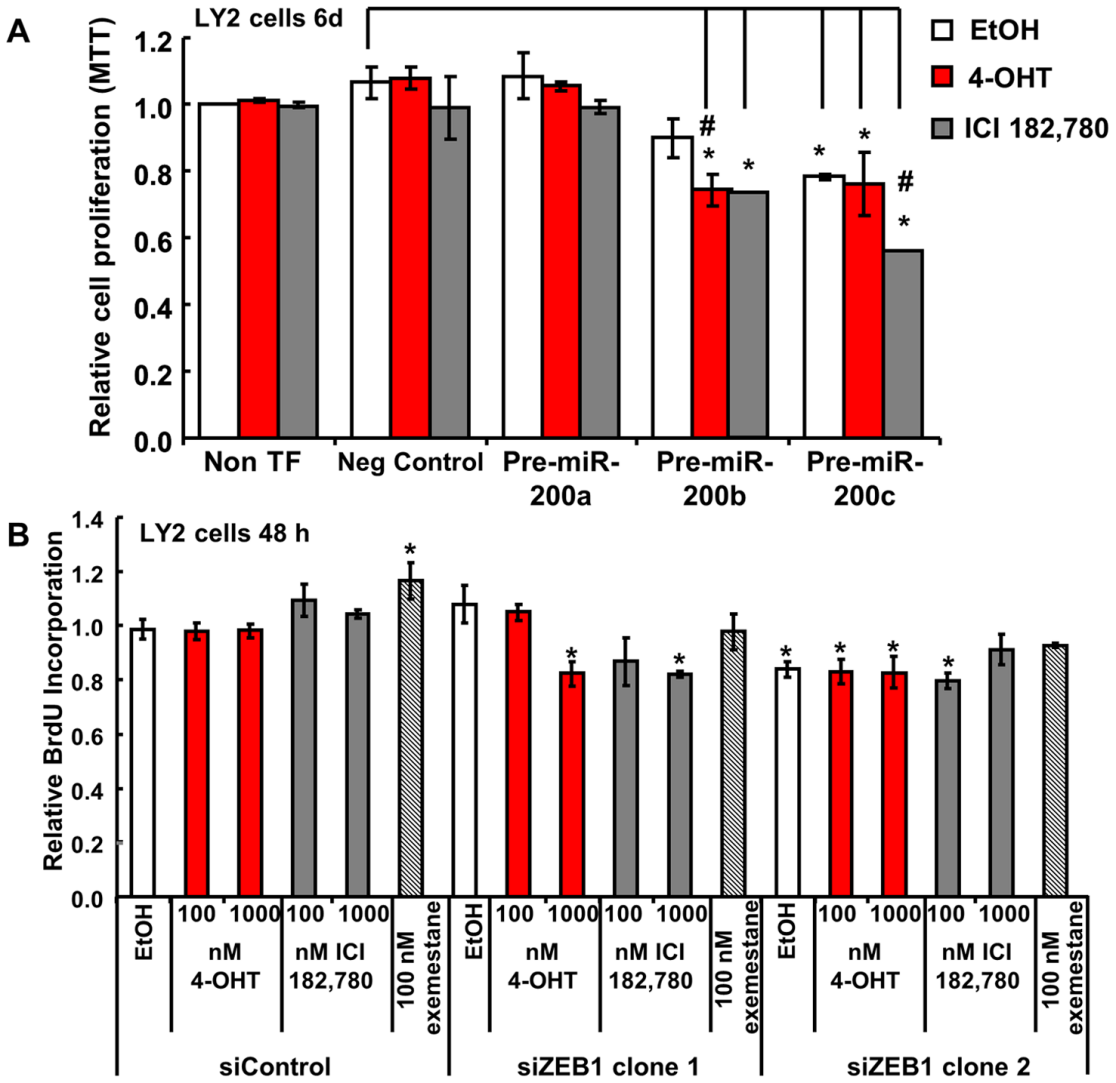
Overexpression of miR-200b or miR-200c or knockdown of ZEB1 enhances sensitivity of LY2 cells to antiestrogens. **A,** LY2 cells were either untransfected (No TF) or transfected with negative control (Neg control) or pre-miR-200a, miR-200b or miR-200c. 5 days post transfection, cells were starved for 24 h and treated with 100 nM 4-OHT or 100 nM fulvestrant for 6 days prior to MTT assay. Values are the mean ± SEM of 4 experiments. *p<0.05 *versus* LY2 EtOH-treated negative control. **B,** LY2 cells were transfected with siRNA negative control (siControl) or two different siZEB1 oligos for 24 h, then treated with the indicated concentrations of 4-OHT, fulvestrant or 100 nM exemestane, an aromatase inhibitor, for 48 h prior to measuring BrdU incorporation. Values are the mean ± SEM of 4 determinations in one experiment. *p<0.05 *versus* LY2 EtOH-treated siControl transfection.

To examine if expression of miR-200 family members affects sensitivity of endocrine-resistant LY2 cells to antiestrogens, cells were transiently transfected with precursors for miR-200a, miR-200b, and miR-200c and MTT cell viability assays were performed in cells treated with vehicle control, 4-OHT, or fulvestrant for 6 days (Fig. 3A). Increased miR-200a, miR-200b and miR-200c expression was confirmed by qPCR even 11 days after transfection, as well as earlier time points (Fig. S2 and data not shown). Treatment of nontransfected or control miRNA-transfected LY2 cells with 4-OHT or fulvestrant had no effect on cell viability (Fig. 3A). LY2 cell viability was unaffected by overexpression of miR-200a regardless of treatment (Fig. 3A). Overexpression of miR-200b increased LY2 cell sensitivity to inhibition by 4-OHT and fulvestrant. Overexpression of miR-200c reduced basal LY2 viability and fulvestrant, but not 4-OHT, further inhibited LY2 viability (Fig. 3).

### ZEB1 Knockdown in LY2 Cells Enhances Inhibition by 4-OHT or Fulvestrant, not Exemestane

Since miR-200 family members repress ZEB1 expression [27], [30] and ZEB1 is expressed in LY2 cells (Fig. 2), we used siRNA to knockdown ZEB1 expression in LY2 cells and examined cell proliferation by BrdU incorporation. Knockdown of ZEB1 was confirmed (Fig. S3). Treatment of control-transfected LY2 cells with 4-OHT, fulvestrant, or exemestane did not affect cell proliferation after 48 h (Fig. 3B). Knockdown of ZEB1 by two different oligonucleotides had slightly different effects: siZEB1 clone 1 had no effect on basal proliferation, and enhanced sensitivity to growth inhibition by 4-OHT and fulvestrant. No effect on exemestane sensitivity was observed, indicating this response is specific to antiestrogen sensitivity. Notably, ZEB1 clone 2 transfection resulted in an overall suppression of basal proliferation. Due to this effect, no further enhancement of antiestrogen sensitivity was observed. These results indicated ZEB1 may play multiple roles in antiestrogen sensitivity and cell survival.

### Inhibition of miR-200b and miR-200c Activity does not Promote Resistance of MCF-7 Cells to Antiestrogens

**Figure 4:**
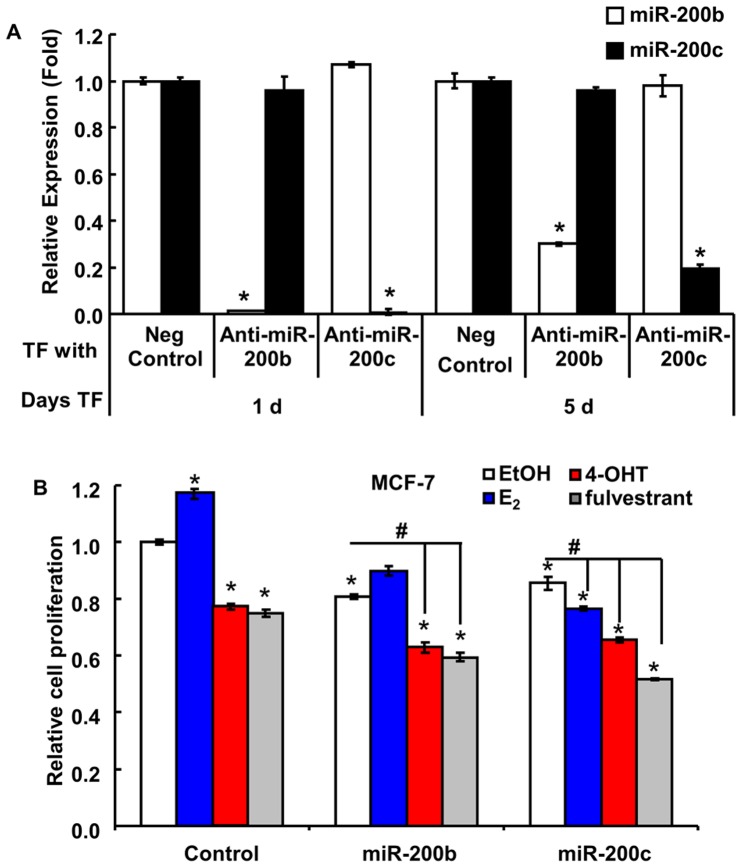
Knockdown of miR-200b or miR-200c does not promote resistance of MCF-7 to 4-OHT or fulvestrant. MCF-7 cells were transfected with a negative control, anti-miR-200b, or anti-miR-200c. 1 d post transfection, cells were treated with 100 nM 4-OHT or 100 nM fulvestrant for 4 d prior to MTT assay. A, CT values for miR-200b and miR-200c in the cells transfected as indicated for 1 or 5 d. Values are the mean ± SEM of 3 determinations. B, MTT values are the mean ± SEM of 3 experiments. *p<0.05 versus EtOH-treated, control-transfected MCF-7 cells; ^#^p<0.05 versus EtOH-treated, miR-200b- or miR-200c- transfected cells.

Converse experiments were performed using anti-miR miRNA inhibitors to bind and inhibit endogenous miR-200b or miR-200c activity in MCF-7 cells. Specific reduction of miR-200b and miR-200c expression was confirmed (Fig. 4A, Fig. S4). Surprisingly, knockdown of miR-200b and miR-200c reduced basal MCF-7 cell viability by ∼15–20% (Fig. 4B). Reduction in miR-200c abrogated the ability of E_2_ to increase cell viability (Fig. 4B). However, there was no further increase in the sensitivity of cells to inhibition by 4-OHT or fulvestrant after knockdown of miR-200b or miR-200c in MCF-7 cells as compared to the effect of 4-OHT and fulvestrant on control-transfected MCF-7 cells (Fig. 4B), indicating that other factors also contribute to the sensitivity of these cells to growth inhibition by antiestrogens. Overexpression of miR-200b or miR-200c partially restores antiestrogen sensitivity to LY2 cells, but other molecules and/or pathways known to be involved in antiestrogen-resistance such as coregulators [40], [41], [42], altered growth factor signaling [43], [44], [45], NFκB activation [46], [47], or other dysregulated microRNAs in addition to the miR-200 family [32] may also be involved in the phenotype of these cells.

### Overexpression of miR-200b or miR-200c Changes LY2 Cell Morphology

**Figure 5:**
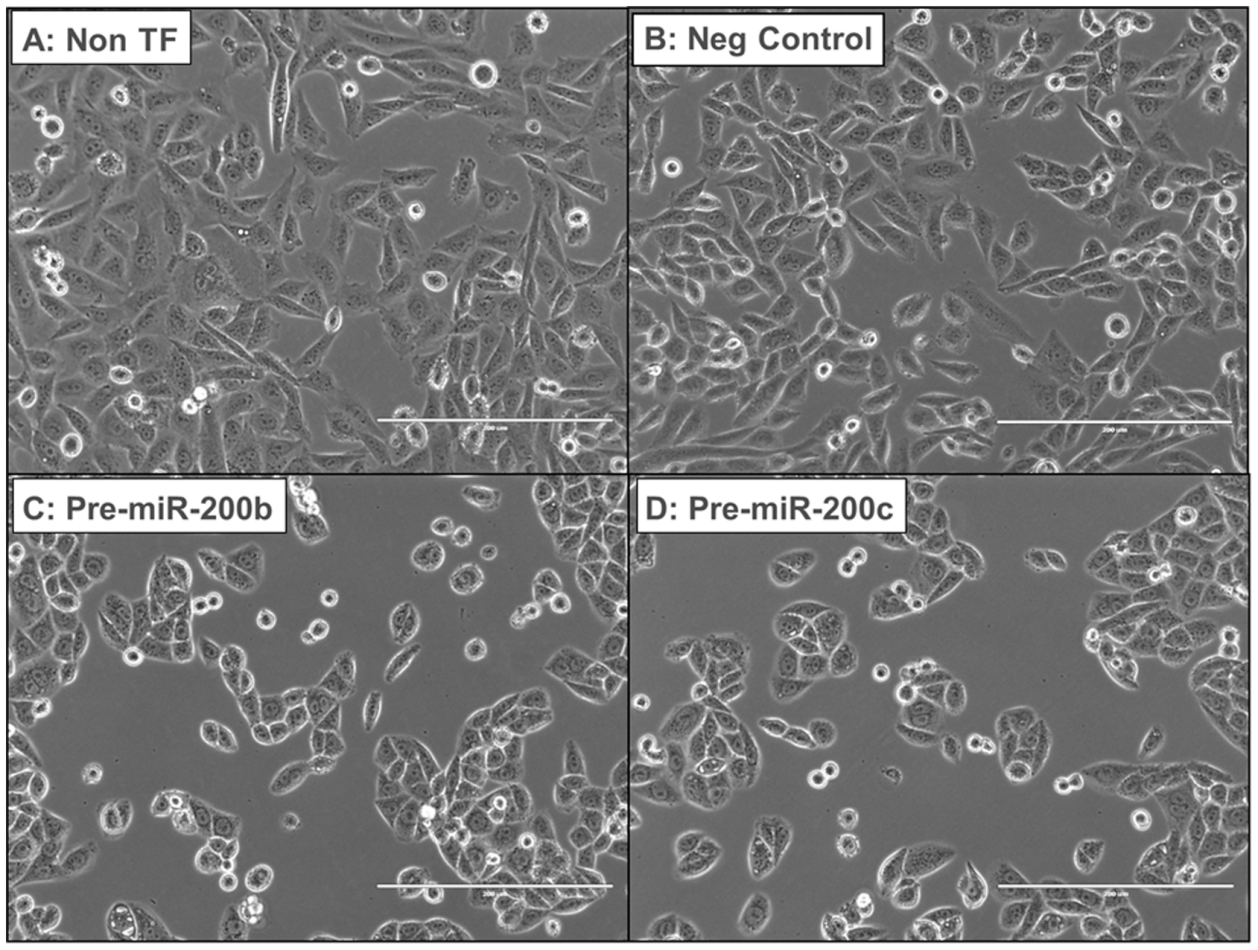
Overexpression of miR-200 family changes LY2 cell morphology from a mesenchymal to an epithelial appearance. LY2 cells were either non-transfected (A), or transfected with control Pre-miR miRNA negative control #1 (Ambion) (B), pre-miR-200b (C), or pre-miR-200c (D) for 72 h. Images of LY2 cells captured using a light microscope (20× magnification, bar = 200 µm).

Since overexpression of miR-200b and miR-200c enhanced antiestrogen-sensitivity of LY2 cells (Fig. 3A), we examined if these miRNAs affected cell morphology. Overexpression of miR-200b and miR-200c, (Fig. S5, Fig. S6), altered LY2 cell morphology (Fig. 5, Fig. S7). The appearance of LY2 cells changed from an elongated/fibroblastic-appearance to a more epithelial or ‘cobble-stone’ shaped appearance with miR-200b and miR-200c transfection (Fig. 5C and D). Overexpression of miR-200a had no effect on LY2 cell appearance (Fig. 5B), in agreement with the lack of effect of miR-200a on cell viability (Fig. 3A). Previous studies reported the reversal of EMT in mesenchymal, triple-negative MDA-MB-231 breast cancer cells with miR-200c overexpression [31]. Our results are in agreement with these data and indicate that LY2 cells assume a more epithelial-like morphology with miR-200b or miR-200c transfection.

### Overexpression of miR-200b or miR-200c in LY2 Reduces ZEB1 and Mesenchymal Markers and Increases E-cadherin

**Figure 6:**
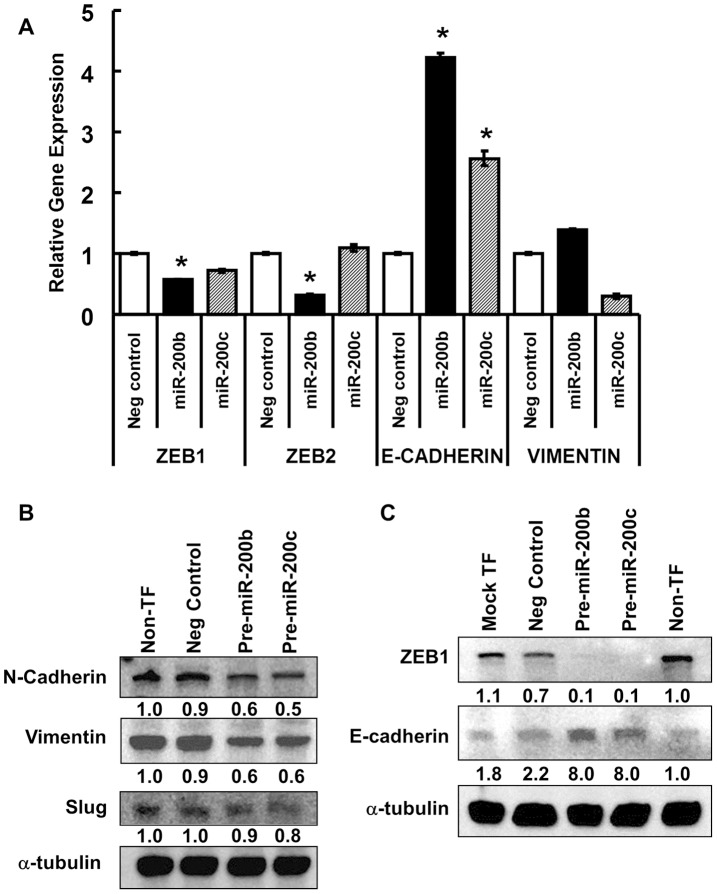
Overexpression of miR-200b and miR-200c inhibits expression of mesenchymal markers and increases E-cadherin in LY2 cells. (A–C) LY2 cells were not transfected (Non-TF), Mock-transfected (RNAiMAX), or transfected with negative control, pre-miR-200b, or pre-miR-200c for 24 h before preparing RNA or WCE for subsequent analysis. A, *ZEB1*, *ZEB2*, E-cadherin (*CDH1*), and vimentin (*VIM*) expression was quantified by qPCR. Values are the mean ± SEM. *p<0.05 versus negative control for each gene. B–C, Protein expression of Zeb1 as well as EMT markers N-cadherin, vimentin, and Slug, and epithelial marker E-cadherin was analyzed by western blotting normalized to the expression of α-tubulin. Non-transfected values are set to one.

To determine if the observed changes in morphology of LY2 cells were due to reduced expression of ZEB1, ZEB2, and mesenchymal markers and increased expression of epithelial marker, ZEB1/2, E-cadherin, and vimentin mRNA expression was examined in LY2 cells overexpressing miR-200b or miR-200c (Fig. 6A). Overexpression of miR-200b and miR-200c reduced ZEB1 and increased E-cadherin (*CDH1*) expression. Overexpression of miR-200b reduced ZEB2 expression while overexpression of miR-200c reduced vimentin (*VIM*) expression (Fig. 6A). At the protein level, miR-200b and miR-200c transfection had the greatest impact on N-cadherin (∼50% reduced expression), while vimentin and Slug were reduced to a lesser extent (Fig. 6B). We observed greatly reduced ZEB1 protein and a concomitant increase in E-cadherin protein in LY2 cells transfected with pre-miR-200b or pre-miR-200c (Fig. 6C). Taken together, these results indicate that reduction of miR-200b and miR-200c contributes to the increase in ZEB1, N-cadherin, vimentin, and Slug and the reduction in E-cadherin in LY2 cells.

### LY2 Cells Overexpressing miR-200b or miR-200c Exhibit Decreased Cell Motility

**Figure 7:**
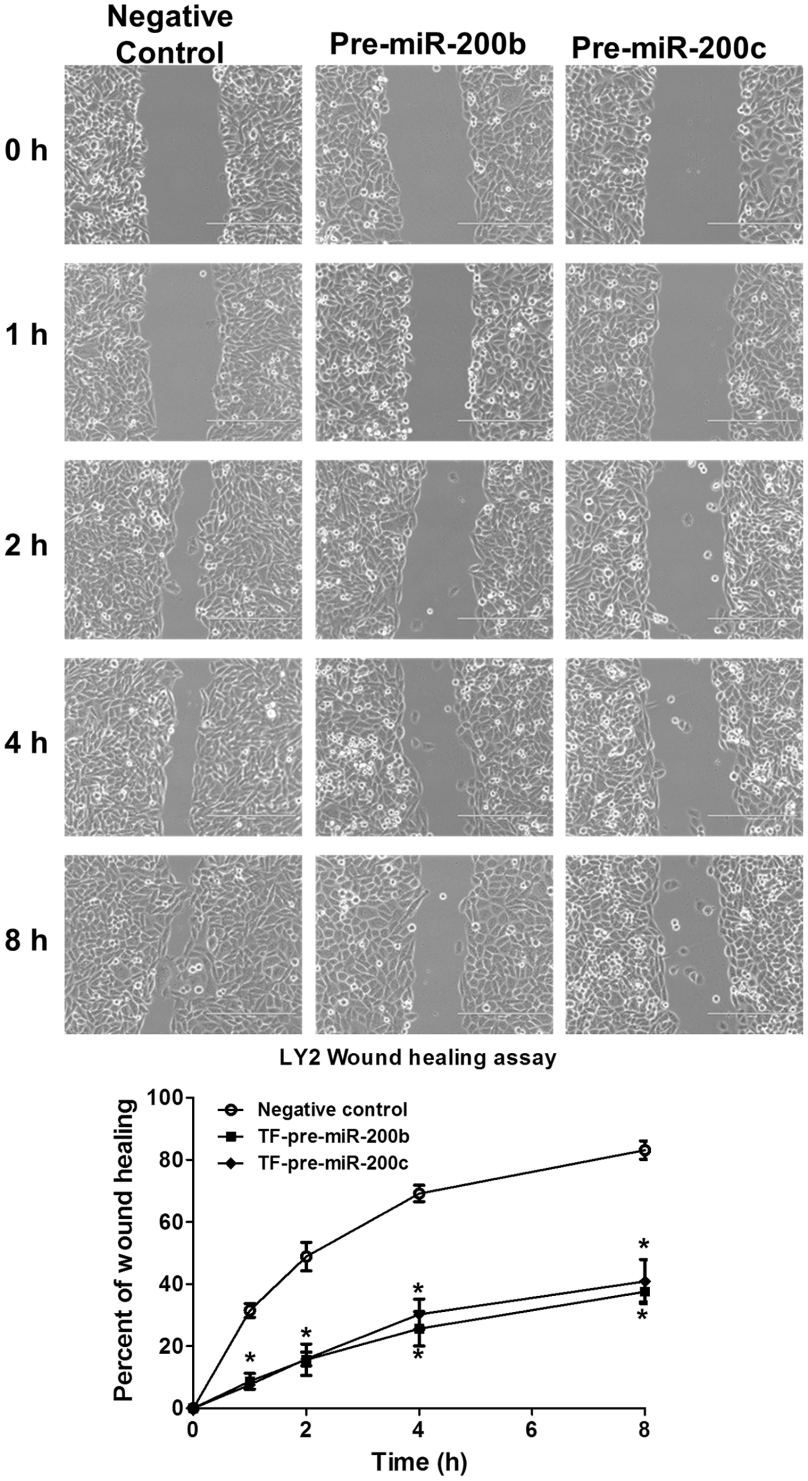
miR-200b and miR-200c inhibit LY2 cell migration in a wound healing assay. LY2 cells were plated in a 6-well plate (3000 cells/well), transfected with negative control, pre-miR-200b, or pre-miR-200c for 24 h. Cells were wounded by scratching using a p200 pipette tip at time zero (0 h). Cells were washed with medium to remove displaced cells. The wound closure was analyzed at the indicated times using NIH Image J software. Values are the mean ± SEM of four separate measurements. *p<0.01 versus negative control-transfected cells. There were no statistical differences between cells transfected with pre-miR-200b versus pre-miR-200c.

To determine if the loss of expression of miR-200b or miR-200c in LY2 affects cell motility, LY2 cells were transiently transfected with a negative control or with pre-miR-200b or pre-miR-200c and cell motility was examined by a wound healing assay (Fig. 7). Overexpression of miR-200b and miR-200c decreased wound healing, a result in agreement with findings in other cell types, *e.g*., miR-200c transfection of BT549 breast cancer and Hec50 endometrial cancer cells [48].

### Inhibitors of Deacetylation and Methylation Increase miR-200 Family Expression in LY2 Cells

**Figure 8:**
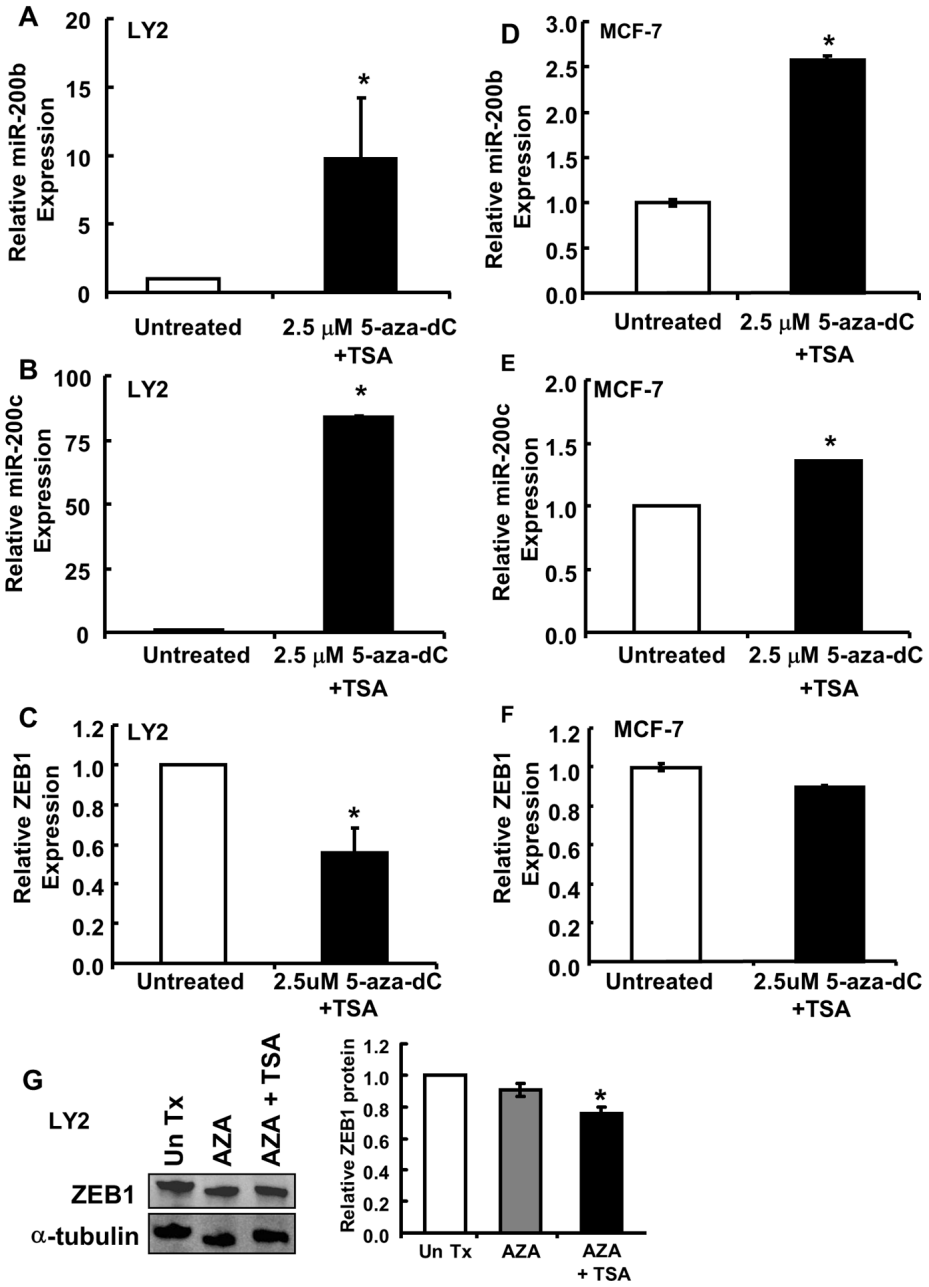
5-aza-dC and TSA increase miR-200b and miR-200c expression in LY2 cells. (A–F) LY2 or MCF-7 cells were treated with 2.5 µM 5-aza-dC for 72 h with 100 ng/ml TSA added for the last 16 h of treatment. The expression of miR-200b, miR-200c or ZEB1 mRNA was determined by qPCR. Values are the mean ± SEM of triplicate determinations. G, LY2 cells were treated with 2.5 µM 5-aza-dC (AZA) alone for 72 h or with 100 ng/ml TSA added for the last 24 h of treatment. Whole cell lysates were separated by 10% SDS-PAGE and western blotted for ZEB1 expression. The membrane was stripped and reprobed for α-tubulin. ZEB1/α-tubulin expression was normalized to non-treated control cells. Values are ± SEM of duplicate determinations. *p<0.05 versus untreated.

Epigenetic changes in chromatin structure may be responsible for reduced expression of miR-200 family members in LY2 cells. Previous studies reported that the CpG island near the miR-200c/miR-141 transcription start site is methylated in fibroblasts and tumors cells that are miR-200c or miR-141-negative [12], [49], [50]. Likewise, methylation of CpG islands in the promoter of the miR-200b cluster was inversely associated with miR-200b expression in breast cancer cells [51]. To determine if decreased expression of miR-200 family members in LY2 cells is due to methylation and histone deacetylation, LY2 cells were treated with 2.5 µM 5-aza-dC alone or in combination with 100 ng/µl TSA, a histone deacetylase (HDAC) inhibitor, for 72 h. TSA was added in the last 16 h of the treatment period [52]. The combined treatment of LY2 cells with 5-aza-dC and TSA increased the expression of miR-200b and miR-200c (Fig. 8A and 8B). Commensurate with reports that miR-200b has a higher percentage of CpG methylation than miR-200c [12], we detected a lower increase in relative miR-200b compared to miR-200c expression in LY2 cells. MCF-7 cells were shown to have relatively low methylation of miR-200b compared to triple negative MDA-MB-231 and BT549 cells [12], but similar methylation of P1 and P2 promoters for miR-200b compared to miR-200b negative MDA-MB-231and MDA-MB-436 cells [51]. Concomitant with the increased expression of miR-200b and miR-200c, there was a decrease in expression of ZEB1 mRNA with 5-aza-dC and TSA treatment (Fig. 8C). We also detected a ∼25% decrease in ZEB1 protein in LY2 cells treated with 5-aza-dC and TSA or TSA alone (Fig. 8G, H). To determine if the observed decrease in ZEB1 mRNA expression is due to a direct effect of the inhibitors, MCF-7 cells were treated with 2.5 µM 5-aza-dC in combination with 100 ng/µl TSA. Combined treatment with 5-aza-dC and TSA increased miR-200b and miR-200c (Fig. 8D, E), but did not alter the expression of ZEB1 in MCF-7 cells (Fig. 8F). Notably, the increase in miR-200b and miR-200c was much less in MCF-7 compared to LY2. These data suggest that methylation and deacetylation play a role in the reduced expression of miR-200b and miR-200c in LY2 cells.

## Discussion

In this study we report novel roles for miR-200b and miR-200c in inhibiting the sensitivity of endocrine-resistant LY2 breast cancer cells to 4-OHT and fulvestrant. We report a progressive decrease in the expression of miR-200a, miR-200b, and miR-200c in an MCF-7-derived cell line model of TAM/endocrine resistance, *i.e*., decreasing from MCF-7, LCC1, LCC2, LCC9, to LY2, respectively. Concurrently, we detected an increase in ZEB1 expression in LCC9 and LY2 cells. Overexpression of miR-200b and miR-200c enhanced the sensitivity of LY2 breast cancer cells to growth inhibition by antiestrogens 4-OHT and fulvestrant. A previous report showed that transfection of MDA-MB-231 cells with pre-miR-200b or pre-miR-200c enhanced their sensitivity to doxorubicin [53], but the data summarized here are the first to indicate roles for miR-200b and miR-200c in antiestrogen sensitivity.

A role for miR-200 family in drug resistance, *i.e*., paclitaxel, was reported in ovarian cancer [54]. Similarly, gemcitabine-resistant pancreatic cancer is associated with decreased miR-200 expression [55]. Resistance of pancreatic cancer cells to gemcitabine was reduced by treatment with natural compounds such curcumin, which increased miR-200 expression [56], [57], [58], [59]. These studies demonstrate that loss of miR-200 has roles in multiple types of drug resistance.

Our results show that there is an inverse relationship between the expression of miR-200 family expression and ZEB1 mRNA in LY2 cells. These data are in agreement with other reports showing an inverse correlation between miR-200 family and ZEB1 expression in basal-like, triple negative breast cancer (TNBC) cells such as MDA-MB-231 and BT549 [14], [28], [30], [31].

LY2 cells overexpressing miR-200b or miR-200c displayed a change in morphology from a spindle-shaped or mesenchymal phenotype to a ‘cobblestone’ or epithelial phenotype (Fig. 5). Cells expressing miR-200a did not show a change in morphology or change in sensitivity to antiestrogens. As miR-200b and miR-200c share the same seed sequence [25], we suggest that the similarity in effects of miR-200b and miR-200c in enhancing antiestrogen-sensitivity and promoting a more epithelial cell morphology may be attributed to common target mRNAs involved in regulating cell morphology, such as genes encoding the actin cytoskeleton associated proteins WAVE3 and MSN (reviewed in [60]), but this speculation will require further research. Other studies reported a reversal of EMT in aggressive breast cancer cell lines transfected with miR-200c or miR-141 [14], [29], [30], [31]. For example, overexpression of miR-200b and miR-200c caused MET in mesenchymal breast cancer cell lines MDA-MB-231 and BT549 by repressing ZEB1 and ZEB2 [27], [30]. Likewise, ectopic expression of miR-200c restored E-cadherin expression and reversed the mesenchymal phenotype in NMuMG (normal murine mammary epithelial cells) and 4TD7 breast carcinoma cells [29]. Our data showing a change in morphology and the decrease in N-cadherin, and to a lesser extent vimentin and Slug, of LY2 cells overexpressing miR-200b and miR-200c are in agreement with these observations, and are concordant with decreased ZEB1 and increased E-cadherin in these cells.

Decreased miR-200 family expression in LY2 cells could be due to epigenetic changes in the promoter, *e.g*., DNA methylation and histone deacetylation. CpG island methylation of miR-200c/miR-141 promoter was reported in breast and prostate cancer cells [49], [50], [61]. Treatment of MDA-MB-231 and BT549 breast and PC3 prostate cancer cells with 5-aza-dC, an inhibitor of DNA methylation, increased miR-200c and miR-141 expression [49]. Our study agrees with these reports of epigenetic silencing of the miR-200 family, because we demonstrated that treatment of LY2 cells with 5-aza-dC+TSA increased miR-200b and miR-200c expression. There was a concomitant decrease in the expression of ZEB1 mRNA and protein.

Endocrine resistance is accompanied by loss of cell-cell adhesion and EMT due to EGFR-mediated phosphorylation and activation of the β-catenin pathway [19] and/or overexpression of the c-Met receptor protein [62]. Further, elevated Src activity contributes to the invasive phenotype of TAM-R MCF-7 cells [63]. Induction of Snail 1 by overexpression of a peptidyl-prolyl isomerase Pin1 which, in turn, activates GSK-3β or NFκB, promotes EMT in MCF-7 TAM-R cells by downregulation of E-cadherin [20]. Taken together, these reports indicate a link between aberrant activation of signaling pathways leading to EMT and endocrine resistance [17]. However, there is only one report of miRNA regulation of both EMT and endocrine-resistance in breast cancer cells [22]. That study showed that overexpression of miR-375 increased sensitivity of TAM-R MCF-7 cells to TAM by decreasing the expression of metadherin (MTDH) which induces EMT in breast cancer cells [22].

Although miR-200 is considered a tumor suppressor miRNA, there are some reports of its role as an oncogene or oncomiR. For example, miR-200 family expression is a marker of poor prognosis and chemoresistance in ovarian cancer [64], [65], [66]. Contrary to the expected decrease in miR-200 expression in metastatic cells, high levels of miR-200b and miR-200c were detected in 4T1 metastatic mouse mammary tumor cells [67]. In concordance, 4T1 cells showed low ZEB1 and high E-cadherin expression. These results indicate that mR-200 has a dual pattern of expression, *i.e*., it suppresses EMT while it promotes metastatic colonization after cells have invaded a distant site. Further, miR-200 had pro-metastatic activity in a mouse model of breast cancer metastasis by targeting Sec23a, a suppressor of metastasis [68]. These studies reflect cell context-specific roles for miR-200 family members that require further study.

**Figure 9:**
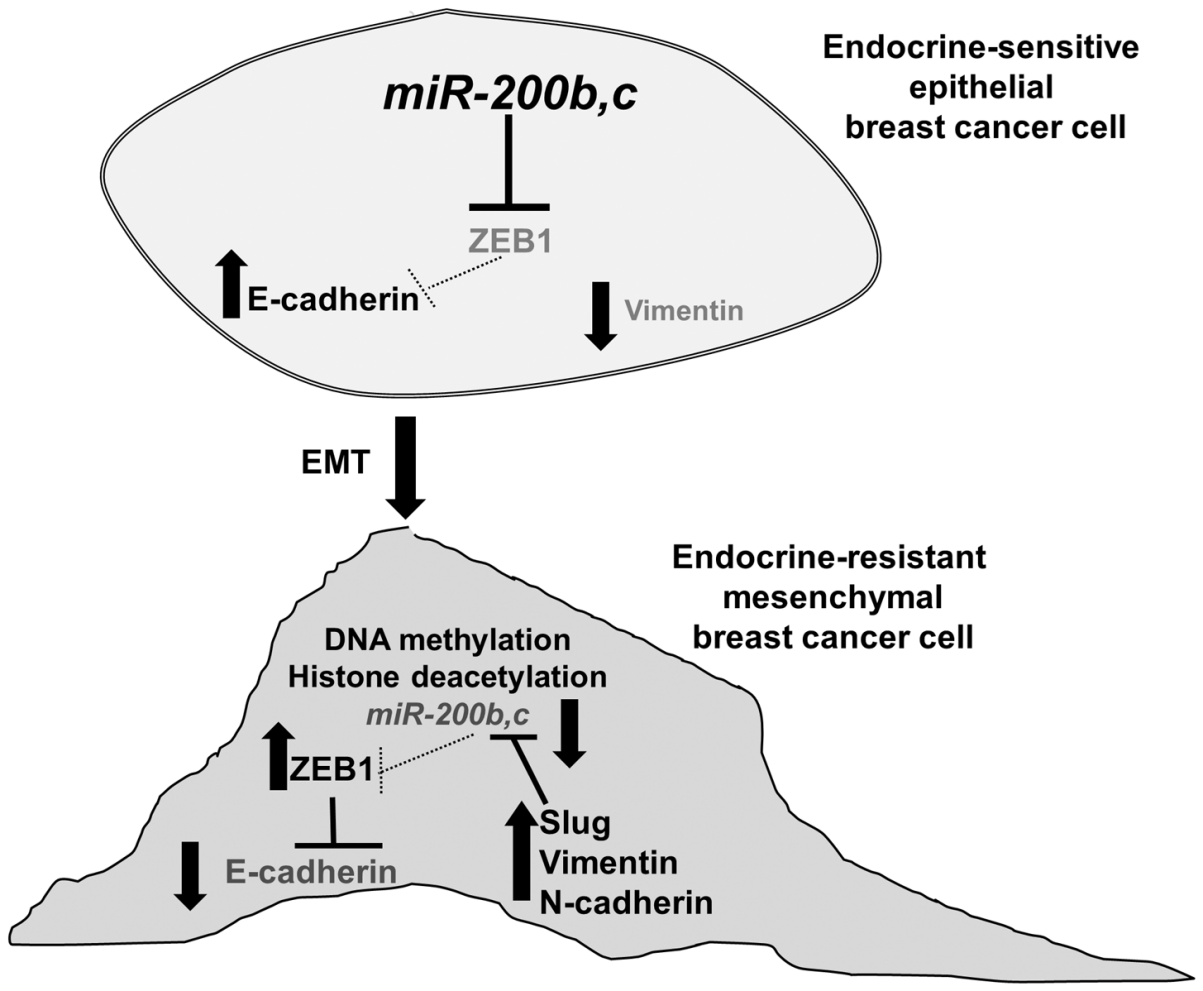
Model of function of miR-200 family members in endocrine resistance in breast cancer cells. A, In endocrine-sensitive breast cancer cells, *e.g*., MCF-7, miR-200b and miR-200c are expressed, resulting in low ZEB1 protein expression. The lack of ZEB1 allows expression of E-cadherin. Vimentin is not expressed. B, In endocrine-resistant cells that have undergone EMT, miR-200 family expression is low, resulting in increased ZEB1 protein which inhibits E-cadherin expression. Vimentin, N-cadherin, and Slug expression is increased. Slug directly inhibits miR-200b expression [69]. Grey lettering indicates reduced expression and dashed lines indicate reduced regulation.

Our results reveal novel roles for miR-200b and miR-200c in conferring antiestrogen sensitivity to endocrine-resistant breast cancer cells (summarized in Fig. 9). In endocrine-sensitive luminal breast cancer cells, expression of miR-200 family members represses ZEB1, thus E-cadherin is expressed and vimentin is repressed and cells have an epithelial phenotype. The decreased miR-200b and miR-200c in endocrine-resistant LY2 cells, caused at least in part by gene methylation and histone deacetylation, results in increased ZEB1 which represses E-cadherin expression, resulting in EMT. The decrease in miR-200b and miR-200c also results in increased N-cadherin, vimentin, and Slug, hallmarks of the mesenchymal phenotype. Although studies have identified a role for miR-200 as a suppressor of EMT, there is little evidence for a role of miR-200 as a suppressor of endocrine resistance in breast cancer cells, hence the novelty of these data. Future experiments are needed to identify targets of miR-200b and miR-200c in antiestrogen sensitivity for targeted therapy.

## Supporting Information

Figure S1 **Effect of E_2_ and 4-OHT on the expression of miR-200 family members in MCF-7, LCC1, LCC2, LCC9, and LY2 cells.** Cells were serum-starved for 48 h and then treated with EtOH (vehicle control), 10 nM E_2_, or 100 nM 4-OHT for 6 h. Values are the mean ± SEM of 3–4 experiments and are expressed as fold relative to EtOH-treated cells. *p<0.05 versus EtOH treated for each cell line.
(TIF)

Figure S2 **Overexpression of miR-200b or miR-200c 11d after transfection of LY2 cells.** LY2 cells were transfected either with pre-miR-200a, pre-miR-200b or pre-miR-200c. RNA was harvested at 11 days and qPCR performed to confirm overexpression of miR-200b or miR-200c. Values are the mean ± SEM of triplicate determinations.
(TIF)

Figure S3 **Knockdown of ZEB1 in LY2 cells.** LY2 cells were transfected with siControl or 2 different clones of siZEB1 or were not transfected (Not TF). 48 h after transfection, RNA was harvested and qPCR for ZEB1 and GAPDH was performed. Values are the average of triplicate determinations ± SEM.
(TIF)

Figure S4 **Knockdown of miR-200b or miR-200c in MCF-7 cells.** MCF-7 cells were transfected with a negative control, anti-miR-200b, or anti-miR-200c and RNA was harvested 1 or 5 d after transfection. CT values for miR-200b and miR-200c in the cells transfected as indicated for 1 or 5 d. Values are the mean ± SEM of 3 determinations.
(TIF)

Figure S5 **Overexpression of miR-200 in transfected cells. LY2 cells were transfected with negative control, pre-miR-200a, pre-miR-200b, or pre-miR-200c.** RNA was harvested at 5 (A) or 7 (B) days after transfection. qPCR performed to confirm overexpression of miR-200a, miR-200b or miR-200c. Values are the mean ± SEM of 3 experiments.
(TIF)

Figure S6 **Overexpression of miR-200 family after 3d of transfection.** LY2 cells were transfected with pre-miR-200a, pre-miR-200b, or pre-miR-200c for 3 d. RNA was harvested at 3 days and qPCR was used to confirm overexpression of miR-200. Values are the mean ± SEM of 3 determinations.
(TIF)

Figure S7 **Overexpression of miR-200 family changes LY2 cell morphology from a mesenchymal to an epithelial appearance.** LY2 cells were transfected with control Pre-miR miRNA negative control #1 (Ambion), pre-miR-200a, pre-miR-200b, or pre-miR-200c for 3 d. A–D. Images of LY2 cells captured using a light microscope (20× magnification, bar- 100 mm scale).
(TIF)
