# Supplementary figures and images for: Reduced Expression of miR-200 Family Members Contributes to Antiestrogen Resistance in LY2 Human Breast Cancer Cells
Source: PLoS One. 2013 Apr 23;8(4):e62334. doi: 10.1371/journal.pone.0062334 (PMC3633860; doi:10.1371/journal.pone.0062334)

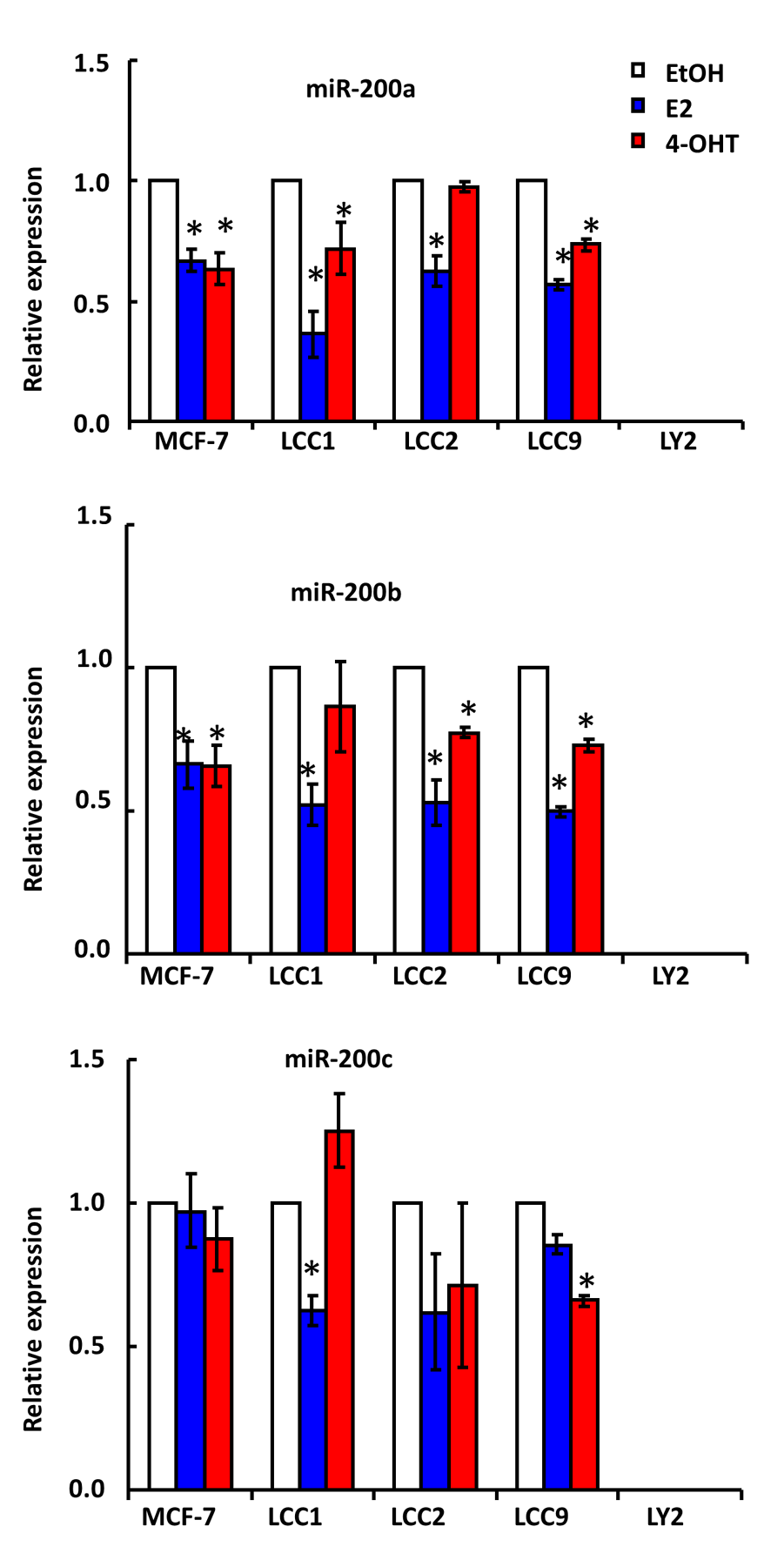

Supplement: Figure S1 — Effect of E2 and 4-OHT on the expression of miR-200 family members in MCF-7, LCC1, LCC2, LCC9, and LY2 cells. Cells were serum-starved for 48 h and then treated with EtOH (vehicle control), 10 nM E2, or 100 nM 4-OHT for 6 h. Values are the mean ± SEM of 3–4 experiments and are expressed as fold relative to EtOH-treated cells. *p<0.05 versus EtOH treated for each cell line. (TIF) [file pone.0062334.s001.tif]
